# 

**Published:** 1904-11

**Authors:** 


					﻿CONTENTS.
ORIGINAL COMMUNICATIONS—
The Stomach-Tube—Its Origin, Use and Abuse. By L, Amster, M.D., Atlanta, Ga ... 505
Georgia Commission on Tuberculosis. By Chas. Hicks, M.D., Dublin, Ga........... 512
Is Tuberculosis a Disease of Environment Only? By H. McHatton, M.D., Macon, Ga.. 518	,
Remarks on Gonorrhea. By W. L. Champion,.M.D., Atlanta, Ga...................... 522	'
SOCIETY REPORTS—
Transactions of the Georgia State Commission on Tuberculosis................... 527
EDITORIAL NOTES AND COMMENTS—
The Georgia State Commission on	Tuberculosis....................................533
The Journal-Record Incorporated............................................... 535.
NEWS AND NOTES....................................................................   533
CONTENTS CONTINUED.................................................................... X
CONTENTS— CONTINUED.
BOOK REVIEWS —
Lectures to General Practitioners on the Diseases of the Stomach and Intes-
tines. (Reed.)........................................................ 539
Von Bergmann’s Surgery. (Bull.)......................................  540
Radiotherapy, Phototherapy, Radium and the High Frequency Currents.
(Allen.)............................................................ 540
Hyde and Montgomery on the Skin....................................... 541
Practical Medicine Series Year Book................................... 541
Beauty Through Hygiene—Common-sense Ways to Health for Girls. (Walker). 542
Suggestive Therapeutics and Forensic Medicine. (Brown and Moyer.)..... 542
SELECTIONS AND ABSTRACTS—
Feeding and the Rest Cure in Typhoid Fever............................ 543
Induction of Premature Labor.......................................... 549
Hygiene of Young Girls................................................ 557
What Should the Medicine-case Hold?................................... 561
Superstitions, Medical and Otherwise.................................. 563
Athletics and Arteriosclerosis........................................ 565
The Fly as a Carrier of Tuberculosis Infection........................ 568
Respiratory Tract: Affections, Symptoms and Treatment................. 569
Cancer of the Breast.................................................. 573
The Post-office Department Suppresses Another Fraud....................574
Appendicitis.........................................................  575
Sir William Roberts on Digestion...................................... 576
MEDICAL ITEMS—Continued...............................xvi, xviii, xx, xxii, xxiv, xxvi
				

